# Commercial fishery disturbance of the global ocean biological carbon sink

**DOI:** 10.1111/gcb.16019

**Published:** 2021-12-18

**Authors:** Emma L. Cavan, Simeon L. Hill

**Affiliations:** ^1^ Department of Life Sciences Imperial College London Ascot Berkshire UK; ^2^ British Antarctic Survey Natural Environment Research Council Cambridge UK

**Keywords:** carbon store, ecosystem services, fisheries, ocean carbon sink

## Abstract

Plankton drive a major sink of carbon across the global oceans. Dead plankton, their faeces and the faeces of plankton feeders, form a huge rain of carbon sinking to the seabed and deep ocean, reducing atmospheric CO_2_ levels and thus helping to regulate the climate. Any change in plankton communities, ecosystems or habitats will perturb this carbon sink, potentially increasing atmospheric CO_2_. Fishing is a major cause of ocean ecosystem disturbance affecting all trophic levels including plankton, but its potential impact on the carbon sink is unknown. As both fisheries and the carbon sink depend on plankton, there is spatial overlap of these fundamental ecosystem services. Here, we provide the first global maps of this spatial overlap. Using an upper quartile analysis, we show that 21% of the total upper ocean carbon sink (export) and 39% of fishing effort globally are concentrated in zones of intensive overlap, representing 9% of the ocean surface area. This overlap is particularly evident in the Northeast Atlantic suggesting this region should be prioritized in terms of research and conservation measures to preserve the high levels of sinking carbon. Small pelagic fish dominate catches here and globally, and their exploitation could reduce important faecal pellet carbon sinks and cause trophic cascades affecting plankton communities. There is an urgent need to recognize that, alongside climate change, fishing might be a critical influence on the ability of the ocean to sequester atmospheric CO_2_. Improved understanding of this influence, and how it will change with the climate, will be important for realizing a sustainable balance of the twin needs for productive fisheries and strong carbon sinks.

## Graphical abstract

Marine life sinks carbon to the deep ocean and sediments, and this can become sequestered—i.e. stored for a long time. The dead material, bodies and faecal pellets from life such as phytoplankton (single‐celled algae), shrimp and fish all contribute to sinking carbon. Fisheries can interupt this carbon sink by resuspending carbon‐rich sediment with their heavy nets, capturing carbon‐sinking species for human or animal food, or by altering the enitre food‐web and affecting the carbon sink. Here we analyse where the highest carbon sink and fishing activity is in the global ocean, and discuss the potential impacts industrial fishing is having on important natural carbon sequestration.

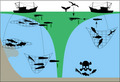

## 1 INTRODUCTION

The ocean biological carbon sink and store (Turner, 2015; Volk & Hoffert, 1985) is an important regulator of atmospheric CO_2_ levels, which would otherwise be 50% higher (Parekh et al., 2006). Estimates of organic carbon exported out of the top 100 m of the global ocean range from 4 to 22 Gt C year^−1^ (Henson et al., 2011; Laws et al., 2000), with an average of 8 Gt C year^−1^ (Saba et al., 2021). Exported carbon sinks down to the deep ocean below the pycnocline where a fraction (~15%, range is temperature dependant and 5%–50% (Marsay et al., 2015; Weber et al., 2016)) is locked away in water masses or sediments on time‐scales from centuries to millennia, and is therefore effectively removed from the carbon cycle, or sequestered (Bax et al., 2020). The rest of the carbon is recycled as it sinks and eventually converted back to CO_2_ by microbes and zooplankton (Turner, 2015). The ~15% of surface carbon export that is sequestered equates to ~1 Gt C year^−1^. For reference, coastal blue carbon sequestration is ~0.38 Gt C year^−1^ or 1.38 Gt CO_2_ year^−1^ (Hoegh‐Guldberg et al., 2019), and anthropogenic carbon release is ~10 Gt C year^−1^ (Zeebe et al., 2016). The ocean biological carbon sink, hereafter ‘carbon sink’, is predominantly driven by phyto‐ and zooplankton at the base of ocean food webs (Turner, 2015). The faecal pellets and carcasses (Halfter et al., 2021) of current and potential fishery species, including anchovy (Saba & Steinberg, 2012), krill (Cavan et al., 2019) and mesopelagic fish (Davison et al., 2013), are also important in sinking carbon and fish are responsible for up to 16% of organic carbon export (Saba et al., 2021). Any marine ecosystem change resulting in deviations in abundance or community composition of species responsible for sinking and storing carbon could result in a positive feedback increasing atmospheric CO_2_ levels (DeVries et al., 2019).

Marine commercial fishing currently removes ~0.10 Gt year^−1^ of biomass (FAO, 2019) and has profoundly altered ecosystems throughout the global ocean. These impacts can propagate through food webs in trophic cascades which produce sequential changes in the abundance of successive trophic levels right down to plankton (Carpenter et al., 1985). Climate change projections predict a decline in the carbon sink due to a shift to smaller plankton communities (Laufkötter et al., 2015), and fishing has already been found to cause similar shifts to smaller plankton (Möllmann et al., 2008). These ecological alterations can affect the lower trophic levels responsible for the majority of carbon fixation, and those that contribute to deeper carbon sinks. Fishing also affects the habitat of the benthos including through the removal of hard substrates such as oyster beds (Grabowski & Peterson, 2007) and the disturbance of sediments which can deplete the deposits of organic material carbon content of sediments (Paradis et al., 2021) and result in remineralization of organic carbon (Luisetti et al., 2019). The reliance of both fish biomass and the carbon sink on phytoplankton (Pauly & Christensen, 1995; Volk & Hoffert, 1985) creates the potential for significant spatial overlap between the two ecosystem services and for fishing to disturb the carbon sink. The potential for fishing to affect the carbon cycles is only beginning to be researched (Bianchi et al., 2021; Mariani et al., 2020; Sala et al., 2021; Trebilco et al., 2020), with a recent modelling study suggesting that fishery‐induced biomass depletion has reduced the global fish community's contribution to carbon export with implications for deep ocean oxygen and nutrient concentrations (Bianchi et al., 2021). Understanding of the impact of past and current fishing on the carbon sink and atmospheric CO_2_ is in its infancy and thus is not acknowledged in fishery management, nor is fishery disturbance factored into forecasts of future changes to the global carbon cycle (Laufkötter et al., 2016). The impact of fishery management practices on climate mitigation needs to be a priority research area (Hoegh‐Guldberg et al., 2019), particularly as countries start to count their natural carbon sinks towards their Nationally Determined Contributions to climate change (Lecerf et al., 2021).

The main reason for the lack of attention to this topic is likely a discipline divide between biogeochemistry and marine ecology. This divide is reflected in models; the biogeochemical modules of the Earth System Models (ESMs) which inform Intergovernmental Panel on Climate Change assessment reports do not include trophic levels above zooplankton (Yool et al., 2013). Whilst ecological modellers are working to better link ESMs and models of fished species (Tittensor et al., 2018), the primary motivation is to investigate the bottom‐up impacts of climate change on these species (Lotze et al., 2019), rather than top‐down controls on the global carbon sink.

We address the potential impact of fishery disturbance on the ocean biological carbon sink. Our aim is to simultaneously map the global export of biological carbon from the surface ocean and fishing intensity, and investigate how the dominant fisheries may disrupt the global carbon sink. We hypothesize that there will be substantial spatial overlap between the two ecosystem services due to their shared reliance on phytoplankton primary production.

**FIGURE 1:**
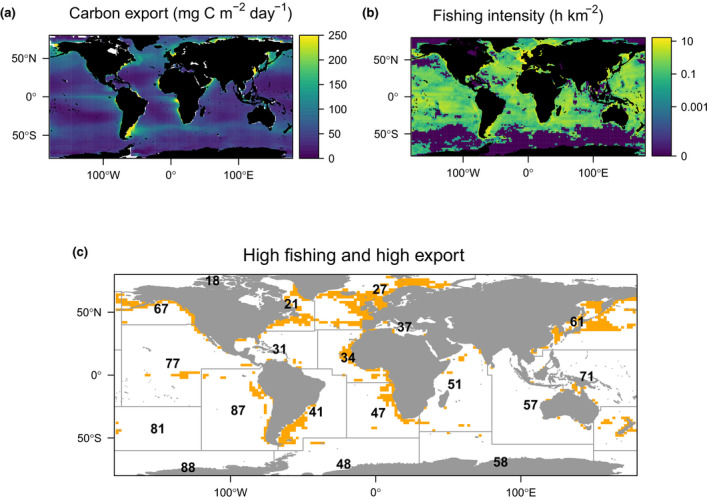
Climatologies of regions of high fishing and carbon export intensity. The mean global annual organic carbon sink (export) computed from four different carbon export algorithms (Figure S1, mg C m^−2^ day^−1^) (DeVries & Weber, 2017; Dunne et al., 2005; Henson et al., 2011; Laws et al., 2011) and fishing intensity as the average annual commercial (vessels 6–146 m in length) fishing on a log *z*‐scale (h km^−2^; Kroodsma et al., 2018). The bottom panel shows where both organic carbon export and fishing intensity are in the upper quartile for both data sets (orange pixels), which is 9% of the surface ocean. Grey grid lines and black numbers indicate the FAO major fishing areas

We use global scale satellite data to assess the spatial overlap between commercial fishing effort (Kroodsma et al., 2018) and the carbon sink (particulate organic carbon, POC, export; DeVries & Weber, 2017; Dunne et al., 2005; Henson et al., 2011; Laws et al., 2011), thereby mapping the risk of fishery disturbance to the carbon sink. Using the mean POC export across four export algorithms (Figure S1) derived from outputs of a satellite‐driven ecosystem model (SIMPLE‐TRIM; DeVries & Weber, 2017), we reproduce annual global POC export with a total of 7.6 Gt C year^−1^ (range 3–10 Gt C year^−1^; Figure 1). This is close to the 8.3 Gt C year^−1^ (range 4–21 Gt C year^−1^) average from 22 different models reported by Saba et al. (2021). We analyse the POC export and fishing data at two scales, namely a 2° × 2° grid and the 19 major UN Food and Agricultural Organisation (FAO) fishing areas (hereafter ‘FAO Area’) used for recording fishery catch statistics. We also identify the routes by which different fishing practices might impact the carbon sink using FAO catch data (FAO, 2019; Table S1).

## 2 SPATIAL OVERLAP OF SINKING CARBON AND FISHING

Both carbon export and fishing intensity are highest around coastlines, which is reflected in the map (orange pixels in Figure 1) showing areas of combined high (upper quartile) carbon export and high fishing intensity. In general, primary production is highest in coastal regions (Behrenfeld & Falkowski, 1997) which also fall within the 200‐nm limit of exclusive economic zones (Kroodsma et al., 2018). The spatial overlap (Figure 1) identified in our upper quartile analysis represents 9% (6%–10%) of the global oceans by area, but 21% (16%–23%) of carbon export and 39% of fishing effort globally. The ranges given correspond to the overlap computed from different export algorithms. The overlap between the two ecosystem services is strongest in the northern hemisphere where land mass, coastlines and human population are greatest.

**FIGURE 2:**
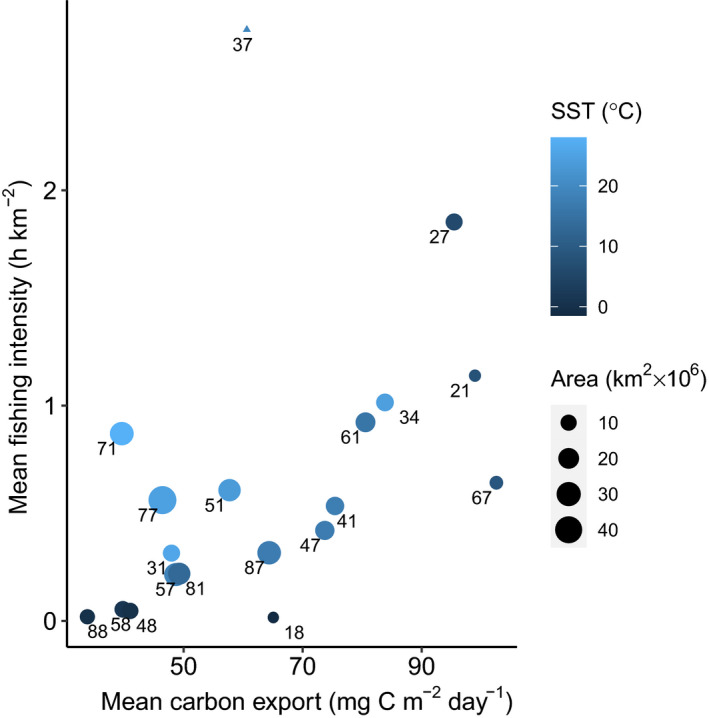
Relationship between carbon export and fishing intensity across FAO areas. The axes show annual mean organic carbon export (mg C m^−2^ day^−1^) and annual fishing intensity (h km^−2^) per unit area (Table 1). Colour of points present the mean sea surface temperature (SST) of each FAO Area and the labels refer to FAO Area number. Size of points indicate the surface area of the FAO Area. Area 37 (Mediterranean) point is a triangle to highlight that the fishing intensity measured for this region is anomalously high (see text). Note the carbon export presented here in the Southern Ocean FAO Areas (48, 58 and 88) is likely an underestimate due to ice and cloud cover masking satellite observations in these regions

The ocean with the greatest overlap of fishing intensity and carbon export is the North Atlantic. The Northeast Atlantic (FAO Area 27) has a high annual mean fishing intensity (1.85 h km^−2^) and high annual mean carbon export (95 mg C m^−2^ day^−1^), even though it covers just 4% of the ocean surface area (Figures 1 and 2). The Northwest Atlantic (FAO Area 21) has similarly high carbon export (99 mg C m^−2^ day^−1^) but a lower annual mean fishing intensity (1.14 h km^−2^) than the Northeast. Other regions of high overlap include the North Pacific (FAO Areas 61 & 67) and the Central Eastern Atlantic (FAO Area 34; Figure 1). Thus, we suggest the North Atlantic, particularly the seas around Europe, followed by the North Pacific need to be prioritized in terms of research to identify how fishing may be impacting the carbon sink. These are also priority areas for the development of measures to minimize fishery disturbance to the sequestration of atmospheric CO_2_ while allowing fishing to continue. A recent global conservation planning framework also identified the Atlantic European Seas as a top priority area for protection due to the high carbon stocks and anthropogenic pressures (Sala et al., 2021). A reduction in the North Atlantic carbon sink caused by climate change has been valued at US$200–3000 billion mostly in mitigation costs (Barange et al., 2017). This indicates the importance of these natural carbon sinks to society. Ideally, any carbon‐related conservation measure would be part of a wider effort of fishery management to restore ecological resilience. Rebuilding of ecosystems that have collapsed or are collapsing in response to exploitation is already an established fisheries management and sustainable development objective (Duarte et al., 2020; Worm et al., 2009), but progress towards this goal is extremely limited (Duarte et al., 2020; Murawski, 2010). Recognizing that the carbon sink is an additional ecosystem service that requires protection strengthens the case for a holistic approach to managing the oceans (Duarte et al., 2020; Long et al., 2015) and might help to achieve a wider suite of environmental goals.

There is a correlation of *r* = .68 (*p* < 0.01), between carbon export and fishing intensity at the FAO Area scale (Figure 2). This correlation excludes the Mediterranean, FAO Area 37, where fishing intensity is anomalously high (2.75 h km^−2^), defined as greater than two standard deviations (2 × 0.69) above the mean (0.66 h km^−2^) for all FAO Areas and attributable to its enclosed geography. The correlation is not causal, but highlights the shared dependency of both carbon export and fishing on primary production. Although sea surface temperature was used to compute carbon export, Figure 2 does not show a monotonic trend with temperature. Rather, both export and fishing are highest in temperate areas and decline towards the subtropics, tropics and Antarctic. Arctic (FAO Area 18) carbon export is close to the mean (60 mg C m^−2^ day^−1^) at 65 mg C m^−2^ day^−1^, but this area has no large‐scale commercial fishing. Nonetheless, melting sea ice and the expiry of the moratorium on central Arctic fishing may change this in the future (Haug et al., 2017). As for the Southern Ocean (FAO Areas 48, 58 and 88), both export and fishing are apparently low according to our analyses, even though the Southern Ocean is an important carbon sink for both organic and inorganic carbon (Cavan et al., 2015; Khatiwala et al., 2009; Laurenceau‐Cornec et al., 2015). In the Atlantic sector of the Southern Ocean, the carbon export can be up to 90 mg C m^−2^ day^−1^ (Cavan et al., 2015). There is some uncertainty in using satellite derived export estimates for this area due to a combination of high levels of cloud cover, ice cover and the importance of larger organisms such as Antarctic krill (Cavan et al., 2019), which are not detected by satellites, although this may change in the near future (Belcher et al., 2021).

## 3 IMPACTS OF FISHING ON THE CARBON SINK

From our analysis of FAO catch data, we identified small and medium (<60 cm length, hereafter ‘small’) pelagic fish as the dominant fished group globally, with trawls the dominant gear type. In the North Atlantic, where fishing intensity and carbon export are high, mackerel and herring dominate the pelagic catch in the east (FAO Area 27), and herring and menhaden dominate in the west (FAO Area 21). Atlantic cod (NE Atlantic) and Northern prawn (NW Atlantic) are the most common groundfish/benthic catch here. The small pelagics, sardines and mackerel dominate the catch in the Central Eastern Atlantic (FAO Area 34) constituting over 50% of the catch. In the North Pacific (FAO Areas 61 & 67), groundfish dominate the catch (Table 1), specifically Alaskan Pollock and Pacific Cod. Japanese anchovy (small pelagics) are the next most fished species in the Northwest Pacific (FAO Area 61).

**TABLE 1:** Carbon export and fishing activity by FAO Area. Data show satellite‐derived annual mean of particulate organic carbon (POC) export per unit area and rank (1 = highest); annual mean fishing intensity per unit area and rank; % of global fishing catch (tonnes year^−1^); and the main fishing gear types and fished groups. Gear type from FAO global capture production data. Main gear types and fished groups cumulatively contributing to ≥50% of catch are reported, with contribution (%) in parentheses. For gear type T, trawl; PS, purse seine; D, dredge; SG, set gillnet; LL, Longline; UG, unknown gear. For species GF, ground; SP, small pelagic; LP, large pelagic; DF, deep water and UF, unspecified fish; PC, pelagic; BC, benthic and UC, unspecified crustaceans; UM, unspecified molluscs; B, bivalves and S, squid

FAO area	Name	POC export		Fishing intensity		Fisheries catch (%)	Main gear type (% catch)	Main fished groups (% catch)
		(mg C m^−2^ day^−1^)	Rank	(h km^−2^)	Rank			
18	Arctic Sea	65.09	8	0.02	19	0	T (36), PS (32)	GF (85)
21	NW Atlantic	98.98	2	1.14	3	2	D (31), T (23)	BC (20), B (12), SP (21)
27	NE Atlantic	95.45	3	1.85	2	11	T (72)*	SP (38), GF (13)
31	Central W Atlantic	47.95	14	0.32	12	2	PS (46), T (23)	SP (36), UF (10), B (5)
34	Central E Atlantic	83.80	4	1.01	4	6	PS (39), T (30)	SP (54)
37	Mediterranean	60.62	10	2.75	1	2	T (38), PS (29)	SP (44), UF (4), B (4)
41	SW Atlantic	75.45	6	0.53	10	2	T (70)	S (21), GF (22), BC (5), DF (4)
47	SE Atlantic	73.75	7	0.42	11	2	T (39), PS (31)	SP (34), GF (19)
48	Antarctic Atlantic	41.04	16	0.05	16	0	T (99)	PC (99)
51	W Indian	57.69	11	0.61	8	6	T (38), SG (22)	UF (14), SP (13), LP (12), GF (8), DF (3), PC (3)
57	E Indian	48.70	13	0.22	14	8	T (33), SG (31)	UF (30), SP (12), LP (3), PC (2), UC (2) GF (2)
58	Antarctic Indian	39.78	17	0.05	17	0	LL (95)	DF (79)
61	NW Pacific	80.57	5	0.92	5	27	T (49), UG (13)	GF (15), SP (14), UF (13), UM (3), PC (3), S (2), LP (2)
67	NE Pacific	102.58	1	0.64	7	4	T (77)	GF (54)
71	Central W Pacific	39.60	18	0.87	6	15	T (45), PS (13)	UF (23), LP (17), SP (8), S (2)
77	Central E Pacific	46.44	15	0.56	9	2	PS (56)	SP (42), LP (9)
81	SW Pacific	49.27	12	0.22	15	1	PS (58)	DF (24), UF (11), SP (14), S (5)
87	SE Pacific	64.37	9	0.32	13	12	PS (78)	SP (57)
88	Antarctic Pacific	33.83	19	0.02	18	0	LL (100)	DF (95)

**FIGURE 3:**
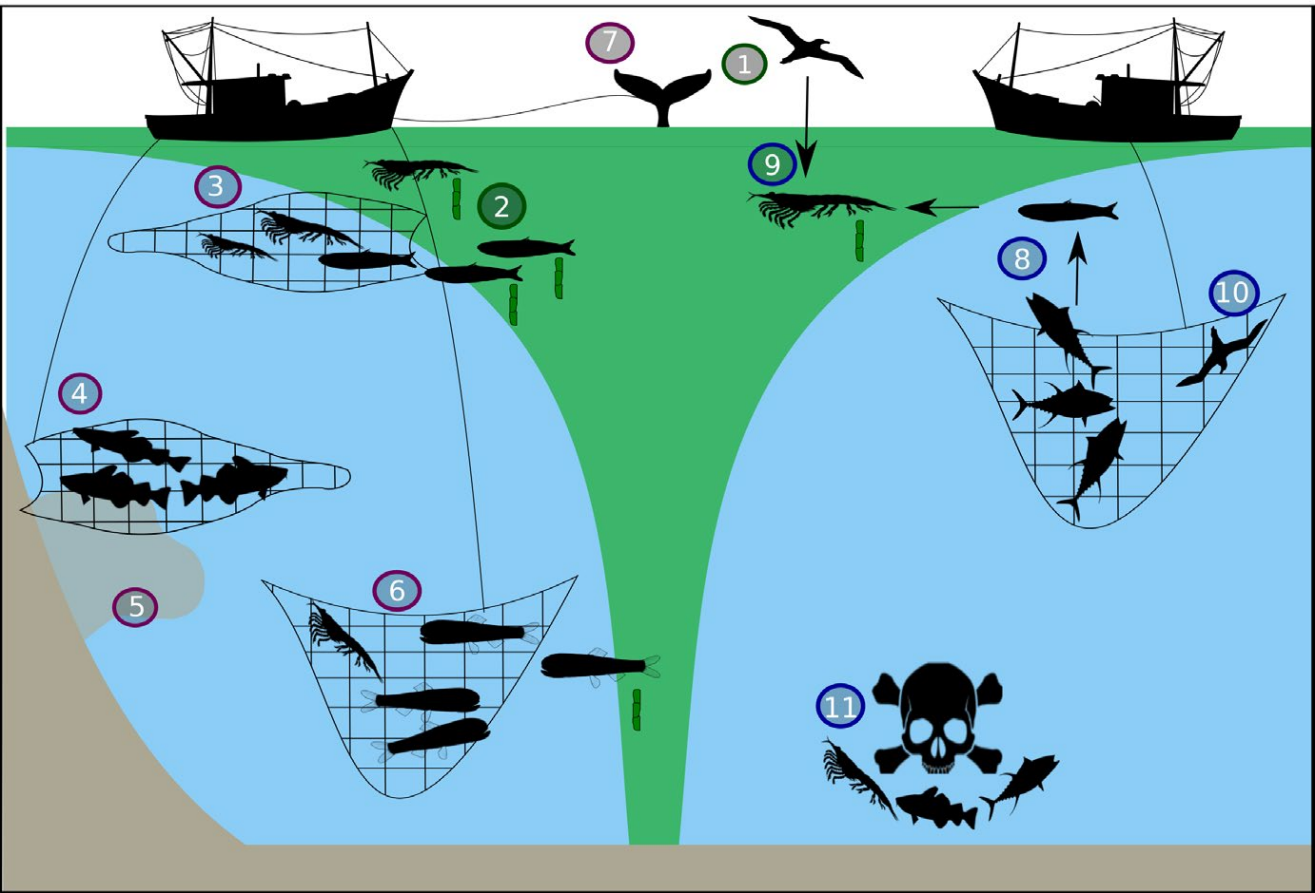
Direct and indirect impacts of fishing to the carbon sink. Phytoplankton (green shading in the surface) stimulate fish biomass production and the export of carbon out of the upper ocean, of which ~15% sinks to the deep ocean. The carbon sink is enhanced by (1) fertilizing species and (2) those egesting fast‐sinking carbon‐rich faecal pellets. Direct impacts of fishing on the carbon sink include (3) harvesting low‐mid trophic level pellet‐producing species, (4) removing species living near the seabed where the sink of carbon will be short, (5) sediment disturbance from groundfish harvesting resulting in resuspension of carbon in the water column and remineralization to CO_2_, (6) removing resident or migratory mesopelagic species that contribute to the carbon sink and finally (7) removing large fish and whales reducing large falls of dead organic matter to the deep sea and sediment. Indirect impacts include (8) causing trophic cascades when removing high trophic level species impacting low trophic level communities that sink carbon, (9) removing prey items for fertilizing species (e.g. mackerel or krill that feed seabirds), (10) killing predators (e.g. seabirds) that may otherwise fertilize the oceans but also help to maintain a balanced food web, and finally (11) the release of discards which could cause localized dead zones

Small pelagic fish contribute to the carbon sink through releasing carbon‐rich faecal pellets that can sink at >1000 m day^−1^ (Saba et al., 2021; Saba & Steinberg, 2012; Figure 3). For example, Peruvian anchoveta may be responsible for around 7% of local carbon export through their faecal pellet sink (Staresinic et al., 1983). Reducing the biomass of these species will reduce the carbon faecal pellet sink, which is one of the most important routes to sink organic carbon (Bisson et al., 2020). Whether the removal of small pelagics indirectly impacts the lower trophic levels such as zooplankton and their faecal sink through trophic cascades remains uncertain. A study in New Zealand showed how fishing just a few species can impact the entire ecosystem, as harvesting snapper and lobster resulted in sea urchins replacing kelp and a change in ecosystem structure and carbon flows (Salomon et al., 2008). Fishing for larger groundfish such as cod in the Baltic Sea increased small pelagic (sprat) biomass, which led to a reduction in its zooplankton prey as part of a more extensive trophic cascade (Casini et al., 2008; Figure 3). Plankton are key drivers of the ocean organic carbon sink (Volk & Hoffert, 1985) and so information on how trophic cascades impact their abundance and composition is required. Depending on factors such as food web structure and the species directly affected by fishing, cascades may have either positive or negative impacts on low trophic level fish and plankton abundance and composition, and thus the carbon sink (Getzlaff & Oschlies, 2017). Regardless, the natural state of a system is best, rather than a human‐altered one which would probably be unstable. Therefore, it is important to evaluate potential trophic cascades caused by fishing major species such as Atlantic herring, mackerel and Japanese anchovy and cod, and how they affect the composition and abundance of carbon exporter communities.

Groundfish such as Atlantic and Pacific cod and Alaska pollock are the next most important catch category after small pelagics (Table 1), but their contribution to the carbon sink is currently unknown. Groundfish fisheries could have the greatest impacts on the carbon sink through trophic cascades as described above in the Baltic Sea (Casini et al., 2008) and physical disturbance of the seabed (Duarte et al., 2020; Luisetti et al., 2019; Pusceddu et al., 2014; Figure 3). The demersal trawls used in these fisheries create plumes of resuspended material that can remove seabed carbon at a rate that counteracts sinking carbon (Pusceddu et al., 2014). A recent study found 30% less organic carbon in deep‐sea (500 m) sediment continuously trawled for shrimp compared to sediment where trawling had been banned for 2 months (Paradis et al., 2021). However, the slow rate of sediment accumulation means a longer ban (decades) on trawling than 2 months is required to restore sediment organic carbon (Paradis et al., 2021). Globally, the amount of CO_2_ released from marine sediments each year due to trawling and dredging is equivalent to 15%–20% of the total CO_2_ absorbed by the ocean each year (Sala et al., 2021), highlighting that marine sediments are one of the most critical carbon reservoirs on the planet (Atwood et al., 2020). As groundfish are mostly found on shallow continental slopes or shelves, they will only contribute to storage of organic carbon in the sediments, and not to inorganic dissolved carbon from respiration in deep waters as open‐ocean deep‐living or migratory fish can. However, the continental shelves store more carbon per unit area (<19,000 Mg km^−2^) than the rest of the ocean provinces including the deep ocean abyssal plains and basins (~6000 Mg km^−2^) due to the higher productivity in the waters above the shelves (Atwood et al., 2020).

As groundfish live close to the sediment, the faecal pellets they egest are subjected to less water column degradation and remineralization prior to the sedimentation of the carbon compared to the pellets of pelagic species. Similarly, mesopelagic fish that live permanently or migrate daily into the mesopelagic zone (200–1000 m) can increase the sink of carbon to the deep sea and seabed (Davison et al., 2013); any carbon they release as faecal pellets or respired CO_2_ below the permanent thermocline (winter mixed layer depth) will not be subjected to water column mixing and remain sequestered for decades or centuries (Boyd et al., 2019; Cavan et al., 2019). The viability of the fisheries for mesopelagic fish is currently being investigated (Grimaldo et al., 2020) and such harvesting is likely to increase the rate at which CO_2_ returns to the atmosphere (Figure 3). The fall of large fishes and mammals (i.e. whales) to the sea floor also transports carbon to the deep sediments, or below the permanent thermocline in the open ocean, and the harvesting of these species reduces the carbon sink further (Haag, 2005; Mariani et al., 2020; Pershing et al., 2010). Other mechanisms by which fishing for any species could impact the carbon sink include the harvesting or by‐catch of fertilizing species such as krill (Schmidt et al., 2016), whales (Ratnarajah et al., 2014) or seabirds (Shatova et al., 2016), rerouting carbon through different trophic cycles, for example, through scavenging seabirds (Votier et al., 2004) and the release of discards (unwanted catch and offal) causing localized dead zones (areas with extremely low levels of dissolved oxygen that can cause mass mortality of most metazoan groups; Figure 3).

Oxygen depletion resulting from the decomposition of fisheries discards has previously been identified as a potential impact of discarding (Clucas, 1997). The major cause of marine dead zones is an enhanced flow of organic material to the seabed which increases microbial respiration (Diaz & Rosenberg, 2008). Localized increases in benthic microbial respiration have been observed as a result of faeces and uneaten food from salmon cages accumulating on the seabed (Findlay et al., 1995). Similarly, the accumulation of gelatinous carcasses as a result of mass die‐offs has led to localized changes in oxygen demands and a switch from autotrophic to heterotrophic systems (Guy‐Haim et al., 2020). While we are not aware of any direct evidence that the release of discards causes dead zones, it is plausible that the accumulation of such matter on the seabed would have a similar, albeit ephemeral and spatially limited, effect.

## 4 CARBON EXPORT VERSUS CARBON SEQUESTRATION

The uncertainties about how fishery disturbance affects the climate extend to the timescales over which particulate carbon exported from the ocean surface returns to the atmosphere. There are many physical and biological processes that can return exported carbon to the ocean surface before it is sequestered in the seabed or deep ocean (Boyd et al., 2019), or can return carbon to the water column after burial in sediments. The majority (~85%) of sinking carbon is consumed and remineralized by heterotrophs such as bacteria, zooplankton or fish. Much of this remineralized carbon will be mixed to the surface and outgassed to the atmosphere (Turner, 2015). True sequestration (sensu Bax et al., 2020) occurs when particulate carbon dissolves and remains in deep water as refractory dissolved organic carbon (Hansell & Carlson, 2013), is buried in anoxic sediments or incorporated into the bodies of long‐lived benthic organisms (Bax et al., 2020). Critically, fish faecal pellets might be one of the most efficient mechanisms for delivering organic carbon to the seabed, equivalent to 10% of the carbon sink (Bianchi et al., 2021). Our analysis highlights the importance of shallow coastal waters where the proximity of the seabed to the surface means faster deposition of exported carbon but also a faster return of carbon to the surface if sediments are disturbed.

## 5 CLIMATE CHANGE, FISHING AND THE CARBON SINK

Global carbon export is projected to decline by the end of the century (Laufkötter et al., 2016) as a result of climate‐driven changes to plankton abundance and composition, and reduced primary production (Laufkötter et al., 2015). Fishing may further exacerbate the decline in carbon export, and thus the store of carbon in the deep ocean, by changing the community composition of low trophic levels important in carbon export. For instance, 30 years of warming in the Baltic Sea changed the dominant copepod species from the larger *Pseudocalanus acuspes* to the smaller *Acartia* spp., with overfishing of cod amplifying this regime shift (Möllmann et al., 2008). Climate change will also likely alter the spatial overlap of fishing and carbon export (Figure 1). Climate‐induced spatial shifts have already been observed in fish, including poleward shifts as sea temperatures rise (Poloczanska et al., 2016). As for the carbon sink, projections suggest an expansion of oligotrophic regions where carbon export is currently low (Figure 1; Bopp et al., 2001), and increases in carbon export towards the poles. Poleward shifts in both fishing intensity and the carbon sink would result in smaller, more concentrated areas of overlap than today (Figure 1), with an increased risk of impact. At present, there are no forecasts of how climate change impacts to higher trophic levels will affect the future carbon sink.

## 6 CONCLUSIONS

We have demonstrated a clear spatial overlap between the carbon sink and commercial fishing. Biomass and ecosystem changes caused by fishing could negatively impact carbon sinking and storage throughout the water column and seabed, and therefore atmospheric CO_2_ levels. Small pelagics are the most commonly fished group, and amongst the most important fish for sinking carbon as faecal pellets. There is an urgent need to clarify through observations and modelling to what extent and how fisheries affect the carbon sink, and to protect this ecosystem service. These needs are particularly important in the regions we identified where the risk of impact is high (North Atlantic). The rebuilding of impacted ecosystems and stocks would help to mitigate this risk. Research is also required into the potential interactions between fisheries disturbance and the effects of climate change on both fishing and the carbon sink. We hope improved understanding of how commercial fisheries affect the carbon sink will be a step towards realizing a sustainable balance of the twin needs for productive fisheries to maintain global food security and strong carbon sinks which play a critical role in climate regulation.

## CONFLICT OF INTEREST

The authors claim no competing interests.

## AUTHOR CONTRIBUTIONS

Emma L. Cavan conceived the study, analysed the carbon export and fishing intensity data and made the figures. Simeon L. Hill analysed the catch and gear type data. Both authors contributed equally to the development of ideas and the writing and editing of this manuscript.

## Supporting information

Supplementary Material

## Data Availability

The data that support the findings of this study are openly available at https://tdevries.eri.ucsb.edu/models‐and‐data‐products/ (SIMPLE_TRIM model output data for climatologies) and https://globalfishingwatch.org/vessel‐tracking‐data/ (fishing intensity data).

## References

[gcb16019-bib-0001] Atwood, T. B. , Witt, A. , Mayorga, J. , Hammill, E. , & Sala, E. (2020). Global patterns in marine sediment carbon stocks. Frontiers in Marine Science, 7. 10.3389/fmars.2020.00165

[gcb16019-bib-0002] Barange, M. , Butenschön, M. , Yool, A. , Beaumont, N. , Fernandes, J. A. , Martin, A. P. , & Allen, J. I. (2017). The cost of reducing the North Atlantic ocean biological carbon pump. Frontiers in Marine Science, 3, 1–10. 10.3389/fmars.2016.00290/full

[gcb16019-bib-0003] Bax, N. , Sands, C. J. , Gogarty, B. , Downey, R. V. , Moreau, C. V. E. , Moreno, B. , Held, C. , Paulsen, M. L. , McGee, J. , Haward, M. , & Barnes, D. K. A. (2021). Perspective: Increasing blue carbon around Antarctica is an ecosystem service of considerable societal and economic value worth protecting. Global Change Biology, 27(1), 5–12. 10.1111/gcb.15392 33064891

[gcb16019-bib-0004] Behrenfeld, M. J. , & Falkowski, P. G. (1997). Photosynthetic rates derived from satellite‐based chlorophyll concentration. Limnology and Oceanography, 42, 1–20.

[gcb16019-bib-0005] Belcher, A. , Fielding, S. , Gray, A. , Biermann, L. , Stowasser, G. , Fretwell, P. , Ireland, L. , & Tarling, G. A. (2021). Experimental determination of reflectance spectra of Antarctic krill (*Euphausia superba*) in the Scotia Sea. Antarctic Science, 33(4), 402–414.

[gcb16019-bib-0006] Bianchi, D. , Carozza, D. A. , Galbraith, E. D. , Guiet, J. , & DeVries, T. (2021). Estimating global biomass and biogeochemical cycling of marine fish with and without fishing. Science Advances, 7(41). 10.1126/sciadv.abd7554 PMC8500507 34623923

[gcb16019-bib-0007] Bisson, K. , Siegel, D. A. , & DeVries, T. (2020). Mechanisms of ocean carbon export in a satellite‐based food web model. Frontiers in Marine Science, 7. 10.3389/fmars.2020.00505

[gcb16019-bib-0008] Bopp, L. , Monfray, P. , Aumont, O. , Dufresne, J.‐L. , Le Treut, H. , Madec, G. , Terray, L. , & Orr, J. C. (2001). Potential impact of climate change on marine export production. Global Biogeochemical Cycles, 15, 81–99. 10.1029/1999GB001256

[gcb16019-bib-0009] Boyd, P. W. , Claustre, H. , Levy, M. , Siegel, D. A. , & Weber, T. (2019). Multi‐faceted particle pumps drive carbon sequestration in the ocean. Nature, 568, 327–335. 10.1038/s41586-019-1098-2 30996317

[gcb16019-bib-0010] Carpenter, S. R. , Kitchell, J. F. , & Hodgson, J. R. (1985). Cascading trophic interactions and lake productivity. BioScience, 35, 634–639. 10.2307/1309989

[gcb16019-bib-0011] Casini, M. , Lövgren, J. , Hjelm, J. , Cardinale, M. , Molinero, J.‐C. , & Kornilovs, G. (2008). Multi‐level trophic cascades in a heavily exploited open marine ecosystem. Proceedings of the Royal Society B: Biological Sciences, 275(1644), 1793–1801. 10.1098/rspb.2007.1752 PMC2587786 18460432

[gcb16019-bib-0012] Cavan, E. L. , Belcher, A. , Atkinson, A. , Hill, S. L. , Kawaguchi, S. , McCormack, S. , Meyer, B. , Nicol, S. , Ratnarajah, L. , Schmidt, K. , Steinberg, D. K. , Tarling, G. A. , & Boyd, P. W. (2019). The importance of Antarctic krill in biogeochemical cycles. Nature Communications, 10(1), 1–13. 10.1038/s41467-019-12668-7 PMC6800442 31628346

[gcb16019-bib-0013] Cavan, E. L. , Laurenceau‐Cornec, E. C. , Bressac, M. , & Boyd, P. W. (2019). Exploring the ecology of the mesopelagic biological pump. Progress in Oceanography, 176, 102125. 10.1016/j.pocean.2019.102125

[gcb16019-bib-0014] Cavan, E. L. , Le Moigne, F. A. C. , Poulton, A. J. , Tarling, G. A. , Ward, P. , Daniels, C. J. , Fragoso, G. M. , & Sanders, R. J. (2015). Attenuation of particulate organic carbon flux in the Scotia Sea, Southern Ocean, is controlled by zooplankton fecal pellets. Geophysical Research Letters, 42, 821–830.

[gcb16019-bib-0015] Clucas, I. (1997). A study of the options for utilization of bycatch and discards from marine capture fisheries. FAO, 59 pp.

[gcb16019-bib-0016] Davison, P. C. , Checkley, D. M. , Koslow, J. A. , & Barlow, J. (2013). Carbon export mediated by mesopelagic fishes in the northeast Pacific Ocean. Progress in Oceanography, 116, 14–30. 10.1016/j.pocean.2013.05.013

[gcb16019-bib-0017] DeVries, T. , Le Quéré, C. , Andrews, O. , Berthet, S. , Hauck, J. , Ilyina, T. , Landschützer, P. , Lenton, A. , Lima, I. D. , Nowicki, M. , Schwinger, J. , & Séférian, R. (2019). Decadal trends in the ocean carbon sink. Proceedings of the National Academy of Sciences of the United States of America, 116, 11646–11651. 10.1073/pnas.1900371116 31138699 PMC6576185

[gcb16019-bib-0018] DeVries, T. , & Weber, T. (2017). The export and fate of organic matter in the ocean: New constraints from combining satellite and oceanographic tracer observations. Global Biogeochemical Cycles, 31(3), 535–555. 10.1002/2016GB005551

[gcb16019-bib-0019] Diaz, R. J. , & Rosenberg, R. (2008). Spreading dead zones and consequences for marine ecosystems. Science, 321(5891), 926–929. 10.1126/science.1156401 18703733

[gcb16019-bib-0020] Duarte, C. M. , Agusti, S. , Barbier, E. , Britten, G. L. , Castilla, J. C. , Gattuso, J.‐P. , Fulweiler, R. W. , Hughes, T. P. , Knowlton, N. , Lovelock, C. E. , Lotze, H. K. , Predragovic, M. , Poloczanska, E. , Roberts, C. , & Worm, B. (2020). Rebuilding marine life. Nature, 580, 39–51. 10.1038/s41586-020-2146-7 32238939

[gcb16019-bib-0021] Dunne, J. P. , Armstrong, R. A. , & Gnanadesikan, A. , & Sarmiento, J. L. (2005). Empirical and mechanistic models for the particle export ratio. Global Biogeochemical Cycles, 19, GB4026.

[gcb16019-bib-0022] FAO (2019). FAO yearbook. Fishery and Aquaculture Statistics 2017.

[gcb16019-bib-0023] Findlay, R. H. , Watling, L. , & Mayer, L. M. (1995). Environmental impact of salmon net‐pen culture on marine benthic communities in Maine: A case study. Estuaries, 18, 145. 10.2307/1352289

[gcb16019-bib-0024] Getzlaff, J. , & Oschlies, A. (2017). Pilot study on potential impacts of fisheries‐induced changes in zooplankton mortality on marine biogeochemistry. Global Biogeochemical Cycles, 31(11), 1656–1673. 10.1002/2017GB005721

[gcb16019-bib-0025] Grabowski, J. H. , & Peterson, C. H. (2007). 15 – Restoring oyster reefs to recover ecosystem services. In K. Cuddington , J. E. Byers , W. G. Wilson , & A. Hastings (Eds.), Ecosystem engineers (pp. 281–298). Academic Press. http://www.sciencedirect.com/science/article/pii/S1875306X07800177

[gcb16019-bib-0026] Grimaldo, E. , Grimsmo, L. , Alvarez, P. , Herrmann, B. , Møen Tveit, G. , Tiller, R. , Slizyte, R. , Aldanondo, N. , Guldberg, T. , Toldnes, B. , Carvajal, A. , Schei, M. , & Selnes, M. (2020). Investigating the potential for a commercial fishery in the Northeast Atlantic utilizing mesopelagic species. ICES Journal of Marine Science, 77(7–8), 2541–2556. 10.1093/icesjms/fsaa114

[gcb16019-bib-0027] Guy‐Haim, T. , Rubin‐Blum, M. , Rahav, E. , Belkin, N. , Silverman, J. , & Sisma‐Ventura, G. (2020). The effects of decomposing invasive jellyfish on biogeochemical fluxes and microbial dynamics in an ultraoligotrophic sea. Biogeosciences, 17, 5489–5511. 10.5194/bg-17-5489-2020

[gcb16019-bib-0028] Haag, A. (2005). Whale fall. Nature, 433(7026), 566–567. 10.1038/433566a 15703715

[gcb16019-bib-0029] Halfter, S. , Cavan, E. L. , Butterworth, P. , Swadling, K. M. , & Boyd, P. W. (2021). “Sinking dead”—How zooplankton carcasses contribute to particulate organic carbon flux in the subantarctic Southern Ocean. Limnology and Oceanography. 10.1002/lno.11971

[gcb16019-bib-0030] Hansell, D. A. , & Carlson, C. A. (2013). Localized refractory dissolved organic carbon sinks in the deep ocean. Global Biogeochemical Cycles, 27(3), 705–710. 10.1002/gbc.20067

[gcb16019-bib-0031] Haug, T. , Bogstad, B. , Chierici, M. , Gjøsæter, H. , Hallfredsson, E. H. , Høines, Å. S. , Hoel, A. H. , Ingvaldsen, R. B. , Jørgensen, L. L. , Knutsen, T. , Loeng, H. , Naustvoll, L.‐J. , Røttingen, I. , & Sunnanå, K. (2017). Future harvest of living resources in the Arctic Ocean north of the Nordic and Barents Seas: A review of possibilities and constraints. Fisheries Research, 188, 38–57. 10.1016/j.fishres.2016.12.002

[gcb16019-bib-0032] Henson, S. A. , Sanders, R. , Madsen, E. , Morris, P. J. , Le Moigne, F. , & Quartly, G. D. (2011). A reduced estimate of the strength of the ocean's biological carbon pump. Geophysical Research Letters, 38, L04606.

[gcb16019-bib-0033] Hoegh‐Guldberg, O. , Caldeira, K. , Chopin, T. , Gaines, S. , Haugan, P. , Hemer, M. , Howard, J. , Konar, M. , Krause‐Jensen, D. , Lovelock, C. E. , Lindstad, E. , Michelin, M. , Nielsen, F. G. , Northrop, E. , Parker, R. W. R. , Roy, J. , Smith, T. , Some, S. , & Tyedmers, P. (2019). The ocean as a solution to climate change: Five opportunities for action. World Resources Institute.

[gcb16019-bib-0034] Khatiwala, S. , Primeau, F. , & Hall, T. (2009). Reconstruction of the history of anthropogenic CO_2_ concentrations in the ocean. Nature, 462(7271), 346–349. 10.1038/nature08526 19924213

[gcb16019-bib-0035] Kroodsma, D. A. , Mayorga, J. , Hochberg, T. , Miller, N. A. , Boerder, K. , Ferretti, F. , Wilson, A. , Bergman, B. , White, T. D. , Block, B. A. , Woods, P. , Sullivan, B. , Costello, C. , & Worm, B. (2018). Tracking the global footprint of fisheries. Science, 359(6378), 904–908. 10.1126/science.aao5646 29472481

[gcb16019-bib-0036] Laufkötter, C. , Vogt, M. , Gruber, N. , Aita‐Noguchi, M. , Aumont, O. , Bopp, L. , Buitenhuis, E. , Doney, S. C. , Dunne, J. , Hashioka, T. , Hauck, J. , Hirata, T. , John, J. , Le Quéré, C. , Lima, I. D. , Nakano, H. , Seferian, R. , Totterdell, I. , Vichi, M. , & Völker, C. (2015). Drivers and uncertainties of future global marine primary production in marine ecosystem models. Biogeosciences, 12, 6955–6984. 10.5194/bg-12-6955-2015

[gcb16019-bib-0037] Laufkötter, C. , Vogt, M. , Gruber, N. , Aumont, O. , Bopp, L. , Doney, S. C. , Dunne, J. P. , Hauck, J. , John, J. G. , Lima, I. D. , Seferian, R. , & Völker, C. (2016). Projected decreases in future marine export production: The role of the carbon flux through the upper ocean ecosystem. Biogeosciences, 13, 4023–4047. 10.5194/bg-13-4023-2016

[gcb16019-bib-0038] Laurenceau‐Cornec, E. C. , Trull, T. W. , Davies, D. M. , Bray, S. G. , Doran, J. , Planchon, F. , & Carlotti, F. (2015). The relative importance of phytoplankton aggregates and zooplankton fecal pellets to carbon export : Insights from free‐drifting sediment trap deployments in naturally iron‐fertilised waters near the Kerguelen Plateau. Biogeosciences, 12(4), 1007–1027.

[gcb16019-bib-0039] Laws, A. , Falkowski, G. , Smith, O. , Hugh, J. , & Mccarthy, J. (2000). Temperature effects on export production in the open ocean. Global Biogeochemical Cycles, 14, 1231–1246.

[gcb16019-bib-0040] Laws, E. A. , Sa, E. D. , & Naik, P. (2011). Simple equations to estimate ratios of new or export production to total production from satellite‐derived estimates of sea surface temperature and primary production. Limnology and Oceanography: Methods, 9(12), 593–601. 10.4319/lom.2011.9.593

[gcb16019-bib-0041] Lecerf, M. , Herr, D. , Thomas, T. , Elverum, C. , Delrieu, E. & Picourt, L. (2021). Coastal and marine ecosystems as Nature‐based Solutions in new or updated Nationally Determined Contributions. https://ocean‐climate.org/wp‐content/uploads/2021/06/coastal‐and‐marine‐ecosystem‐2806.pdf

[gcb16019-bib-0042] Long, R. D. , Charles, A. , & Stephenson, R. L. (2015). Key principles of marine ecosystem‐based management. Marine Policy, 57, 53–60. 10.1016/j.marpol.2015.01.013

[gcb16019-bib-0043] Lotze, H. K. , Tittensor, D. P. , Bryndum‐buchholz, A. , Eddy, T. D. , & Cheung, W. W. L. (2019). Global ensemble projections reveal trophic amplification of ocean biomass declines with climate change. Proceedings of the National Academy of Sciences of the United States of America, 116(26), 12907–12912. 10.1073/pnas.1900194116 31186360 PMC6600926

[gcb16019-bib-0044] Luisetti, T. , Turner, R. K. , Andrews, J. E. , Jickells, T. D. , Kröger, S. , Diesing, M. , Paltriguera, L. , Johnson, M. T. , Parker, E. R. , Bakker, D. C. E. , & Weston, K. (2019). Quantifying and valuing carbon flows and stores in coastal and shelf ecosystems in the UK. Ecosystem Services, 35, 67–76. 10.1016/j.ecoser.2018.10.013

[gcb16019-bib-0045] Mariani, G. , Cheung, W. W. L. , Lyet, A. , Sala, E. , Mayorga, J. , Velez, L. , Gaines, S. D. , Dejean, T. , Troussellier, M. , & Mouillot, D. (2020). Let more big fish sink: Fisheries prevent blue carbon sequestration—Half in unprofitable areas. Science Advances, 6, eabb4848. 10.1126/sciadv.abb4848 33115738 PMC7608781

[gcb16019-bib-0046] Marsay, C. , Sanders, R. , Henson, S. , Pabortsava, K. , Achterberg, E. , & Lampitt, R. (2015). Attenuation of sinking particulate organic carbon flux through the mesopelagic ocean. Proceedings of the National Academy of Sciences of the United States of America, 12, 1089–1094. 10.1073/pnas.1415311112 PMC4313834 25561526

[gcb16019-bib-0047] Möllmann, C. , Müller‐Karulis, B. , Kornilovs, G. , & St John, M. A. (2008). Effects of climate and overfishing on zooplankton dynamics and ecosystem structure: Regime shifts, trophic cascade, and feedback loops in a simple ecosystem. ICES Journal of Marine Science, 65, 302–310. 10.1093/icesjms/fsm197

[gcb16019-bib-0048] Murawski, S. A. (2010). Rebuilding depleted fish stocks: The good, the bad, and mostly, the ugly. ICES Journal of Marine Science, 67, 1830–1840. 10.1093/icesjms/fsq125

[gcb16019-bib-0049] Paradis, S. , Goni, M. , Masque, P. , Duran, R. , Arjona‐Camas, M. , Palanques, A. , & Puig, P. (2021). Persistence of biogeochemical alterations of deep‐sea sediments by bottom trawling. Geophysical Research Letters, 48(2). 10.1029/2020GL091279

[gcb16019-bib-0050] Parekh, P. , Dutkiewicz, S. , Follows, M. J. , & Ito, T. (2006). Atmospheric carbon dioxide in a less dusty world. Geophysical Research Letters, 33. 10.1029/2005GL025098

[gcb16019-bib-0051] Pauly, D. , & Christensen, V. (1995). Primary production required to sustain global fisheries. Nature, 374(6519), 255–257. 10.1038/374255a0

[gcb16019-bib-0052] Pershing, A. J. , Christensen, L. B. , Record, N. R. , Sherwood, G. D. , & Stetson, P. B. (2010). The impact of whaling on the ocean carbon cycle: Why bigger was better. PLoS One, 5, 1–9. 10.1371/journal.pone.0012444 PMC2928761 20865156

[gcb16019-bib-0053] Poloczanska, E. S. , Burrows, M. T. , Brown, C. J. , García Molinos, J. , Halpern, B. S. , Hoegh‐Guldberg, O. , Kappel, C. V. , Moore, P. J. , Richardson, A. J. , Schoeman, D. S. , & Sydeman, W. J. (2016). Responses of marine organisms to climate change across oceans. Frontiers in Marine Science, 3. 10.3389/fmars.2016.00062

[gcb16019-bib-0054] Pusceddu, A. , Bianchelli, S. , Martín, J. , Puig, P. , Palanques, A. , Masqué, P. , & Danovaro, R. (2014). Chronic and intensive bottom trawling impairs deep‐sea biodiversity and ecosystem functioning. Proceedings of the National Academy of Sciences of the United States of America, 111(24), 8861–8866. 10.1073/pnas.1405454111 24843122 PMC4066481

[gcb16019-bib-0055] Ratnarajah, L. , Bowie, A. R. , Lannuzel, D. , Meiners, K. M. , & Nicol, S. (2014). The biogeochemical role of baleen whales and krill in southern ocean nutrient cycling. PLoS One, 9(12), 1–18. 10.1371/journal.pone.0114067 PMC4254789 25469984

[gcb16019-bib-0056] Saba, G. K. , Burd, A. B. , Dunne, J. P. , Hernández‐León, S. , Martin, A. H. , Rose, K. A. , Salisbury, J. , Trueman, C. N. , Wilson, R. W. , & Wilson, S. E. (2021). Toward a better understanding of fish‐based contribution to ocean carbon flux. Limnology and Oceanography, 66(5), 1639–1664. 10.1002/lno.11709

[gcb16019-bib-0057] Saba, G. K. , & Steinberg, D. K. (2012). Abundance, composition and sinking rates of fish fecal pellets in the Santa Barbara Channel. Scientific Reports, 2(1). 10.1038/srep00716 PMC3466448 23056908

[gcb16019-bib-0058] Sala, E. , Mayorga, J. , Bradley, D. , Cabral, R. B. , Atwood, T. B. , Auber, A. , Cheung, W. , Costello, C. , Ferretti, F. , Friedlander, A. M. , Gaines, S. D. , Garilao, C. , Goodell, W. , Halpern, B. S. , Hinson, A. , Kaschner, K. , Kesner‐Reyes, K. , Leprieur, F. , McGowan, J. , … Lubchenco, J. (2021). Protecting the global ocean for biodiversity, food and climate. Nature, 592, 397–402. 10.1038/s41586-021-03371-z 33731930

[gcb16019-bib-0059] Salomon, A. K. , Shears, N. T. , Langlois, T. J. , & Babcock, R. C. (2008). Cascading effects of fishing can alter carbon flow through a temperate coastal ecosystem. Ecological Applications, 18(8), 1874–1887. 10.1890/07-1777.1 19263885

[gcb16019-bib-0060] Schmidt, K. , Schlosser, C. , Atkinson, A. , Fielding, S. , Venables, H. J. , Waluda, C. M. , & Achterberg, E. P. (2016). Zooplankton gut passage mobilizes lithogenic iron for ocean productivity. Current Biology, 26, 2667–2673. 10.1016/j.cub.2016.07.058 27641768

[gcb16019-bib-0061] Shatova, O. , Wing, S. R. , Gault‐Ringold, M. , Wing, L. , & Hoffmann, L. J. (2016). Seabird guano enhances phytoplankton production in the Southern Ocean. Journal of Experimental Marine Biology and Ecology, 483, 74–87.

[gcb16019-bib-0062] Staresinic, N. , Farrington, J. , Gagosian, R. B. , Clifford, C. H. , & Hulburt, E. M. (1983). Downward transport of particulate matter in the Peru coastal upwelling: Role of the anchoveta, *Engraulis ringens* . In E. Suess , & J. Thiede (Eds.), Coastal upwelling its sediment record (Vol. 10B). NATO Conference Series (IV Marine Sciences). Springer. 10.1007/978-1-4615-6651-9_12

[gcb16019-bib-0063] Tittensor, D. P. , Eddy, T. D. , Lotze, H. K. , Galbraith, E. D. , Cheung, W. , Barange, M. , Blanchard, J. L. , Bopp, L. , Bryndum‐Buchholz, A. , Büchner, M. , Bulman, C. , Carozza, D. A. , Christensen, V. , Coll, M. , Dunne, J. P. , Fernandes, J. A. , Fulton, E. A. , Hobday, A. J. , & Huber, V. , … Walker, N. D. (2018). A protocol for the intercomparison of marine fishery and ecosystem models: Fish‐MIP v1. 0. Geoscientific Model Development, 11, 1421–1442. 10.5194/gmd-11-1421-2018

[gcb16019-bib-0064] Trebilco, R. , Melbourne‐Thomas, J. , & John, A. (2020). The policy relevance of Southern Ocean food web structure : Implications of food web change for fisheries, conservation and carbon sequestration. Marine Policy, 115, 103832. 10.1016/j.marpol.2020.103832

[gcb16019-bib-0065] Turner, J. T. (2015). Zooplankton fecal pellets, marine snow, phytodetritus and the ocean's biological pump. Progress in Oceanography, 130, 205–248. 10.1016/j.pocean.2014.08.005

[gcb16019-bib-0066] Volk, T. , & Hoffert, M. (1985). Ocean carbon pumps: Analysis of relative strengths and efficiencies in ocean‐driven atmospheric CO_2_ changes. In E. T. Sundquist , & W. S. Broecker (Eds.), The carbon cycle and atmospheric CO_2_: Natural variations archean to present (pp. 99–110). American Geophysical Union.

[gcb16019-bib-0067] Votier, S. C. , Furness, R. W. , Bearhop, S. , Crane, J. E. , Caldow, R. W. G. , Catry, P. , Ensor, K. , Hamer, K. C. , Hudson, A. V. , Kalmbach, E. , Klomp, N. I. , Pfeiffer, S. , Phillips, R. A. , Prieto, I. , & Thompson, D. R. (2004). Changes in fisheries discard rates and seabird communities. Nature, 427, 727–730. 10.1038/nature02315 14973483

[gcb16019-bib-0068] Weber, T. , Cram, J. A. , Leung, S. W. , DeVries, T. , & Deutsch, C. (2016). Deep ocean nutrients imply large latitudinal variation in particle transfer efficiency. Proceedings of the National Academy of Sciences of the United States of America, 113, 8606–8611. 10.1073/pnas.1604414113 27457946 PMC4978250

[gcb16019-bib-0069] Worm, B. , Hilborn, R. , Baum, J. K. , Branch, T. A. , Collie, J. S. , Costello, C. , Fogarty, M. J. , Fulton, E. A. , Hutchings, J. A. , Jennings, S. , Jensen, O. P. , Lotze, H. K. , Mace, P. M. , McClanahan, T. R. , Minto, C. , Palumbi, S. R. , Parma, A. M. , Ricard, D. , Rosenberg, A. A. , … Zeller, D. (2009). Rebuilding global fisheries. Science, 325(5940), 578–585. 10.1126/science.1173146 19644114

[gcb16019-bib-0070] Yool, A. , Popova, E. E. , & Anderson, T. R. (2013). MEDUSA‐2.0: An intermediate complexity biogeochemical model of the marine carbon cycle for climate change and ocean acidification studies. Geoscientific Model Development, 6, 1767–1811.

[gcb16019-bib-0071] Zeebe, R. E. , Ridgwell, A. , & Zachos, J. C. (2016). Anthropogenic carbon release rate unprecedented during the past 66 million years. Nature Geoscience, 9, 325–329. 10.1038/ngeo2681

