# Supplementary material for: Commercial fishery disturbance of the global ocean biological carbon sink
Source: Glob Chang Biol. 2021 Dec 18;28(4):1212–21. doi: 10.1111/gcb.16019 (PMC9300016; doi:10.1111/gcb.16019)
Supplement: Supplementary file 1 — Supplementary Material [file GCB-28-1212-s001.pdf]

Supplementary Information:

Commercial fishery disturbance of the global ocean biological carbon sink

Cavan, E. L[1]. & Hill, S. L.[2]

[1]Imperial College London, Silwood Park Campus, Ascot, Berkshire, SL5 7PY, UK.
[2]British Antarctic Survey, Natural Environment Research Council, High Cross, Madingley Rd, Cambridge, CB3 0ET, UK.

Contents

1. Supplementary Methods, Global data analysis, Fig. S1
2. Supplementary Methods, Information on catch categories, Table S1
3. Supplementary Methods, Indicators of carbon sink intensity and fishing intensity, Fig. S2
4. References

Supplementary Methods

1. Global data analysis

The current study uses global scale satellite data to assess the spatial overlap between commercial fishing effort (Kroodsma *et al.*, 2018) and the carbon sink (specifically particulate organic carbon, POC, export), thereby mapping the risk of impact. We analyse these data at two scales, namely a 2° x 2° grid and the nineteen major fishing areas (hereafter 'FAO Areas') used by the UN Food and Agricultural Organisation (FAO) for recording catch statistics. We also identify the routes by which different fishing practices might impact the carbon sink.

We used fishing intensity data (hours fished km^{-2}) for the years 2012 – 2016, which is published online by the Global Fishing Watch (Kroodsma *et al.*, 2018) and covers all vessels with an automatic identification system (AIS).

Four different carbon export ratio algorithms were used to determine the mean POC export out of the surface ocean globally (Dunne *et al.*, 2005; Henson *et al.*, 2011; Laws *et al.*, 2011; DeVries and Weber, 2017). Data were taken from the SIMPLE_TRIM model output (DeVries and Weber, 2017), which includes a climatology for net primary production, euphotic zone depth and temperature from satellite data for the years 1997-2008 on a 2° x 2° grid. See individual papers for the export calculations. The total export globally per year ranged from 3.2 Gt C yr^{-1} from the Henson et al. 2011 algorithm, to 10.2 Gt C yr^{-1} from the Dunne et al. 2005 algorithm (Fig. S1).

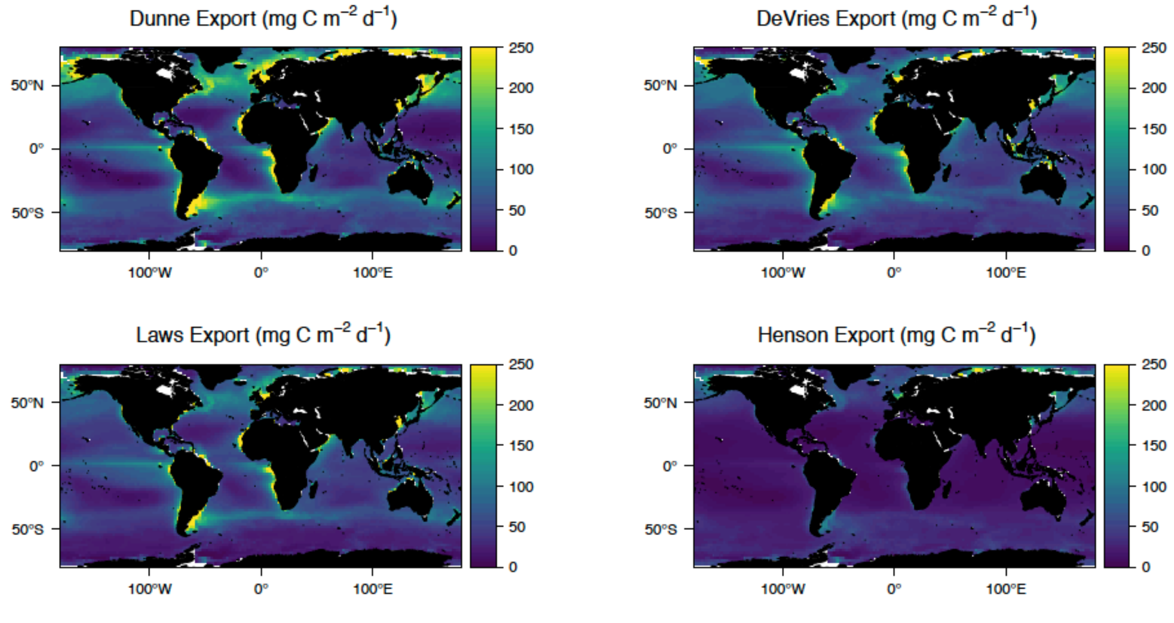

Fig. S1. POC export from the upper ocean from four different algorithms. *The mean POC export from all four algorithms was used to map the overlap of POC and fishing in this study.*

Both data sets (fishing intensity and mean POC export) were represented on a 2° x 2° grid. The area of these grid cells varies with latitude. Our mean values per unit area within each FAO Area (Table 1, Fig. 2) are therefore weighted by the areas of the individual grid cells. To allow comparison between different sized FAO Areas, we provide estimates of fishing

intensity (F_int, hours km^{-2}) and carbon export (C_exp, mg C m^{-2} d^{-1}) per unit area, calculated as follows:

$$F\_int_j = \frac{\sum_{i=1}^{n}\left(F\_int_{ij} * Area_{ij}\right)}{\sum_{i=1}^{n} Area_{ij}}$$

$$C\_exp_j = \frac{\sum_{i=1}^{n}\left(C\_exp_{ij} * Area_{ij}\right)}{\sum_{i=1}^{n} Area_{ij}}$$

where $Area_{ij}$ is the area of the ith of n pixels in the jth FAO area.

2. Information on catch categories and gear types

We obtained gear type data primarily from Tanocet et al. (2019), which provides total Global Fisheries Landings database (Watson, 2017) effort by gear type for 2010 to 2014. We also obtained catch data for the equivalent period from the FAO Global Capture Production database (FAO, 2019) (Table S1). This period overlaps our fishing intensity data for three years, 2012 – 2014, and fishery catch and effort data are well correlated (Fig. S2). Each of these data sources provides aggregated information by FAO area.

The Global Capture Production database provides catch information by taxon. In most cases taxon is resolved to species, but in some cases it is resolved to a lower taxonomic level (e.g. "sardinellas" or "various squids"). We identified the taxa which dominate the catch in each FAO Area (i.e. the top ranking taxa in terms of catch weight, which collectively comprise 50 % or the closest value above 50 % of the overall catch) (Table 1). This resulted in a list of 62 species which we assigned to one of ten categories of catch species (Table S1) on the basis of the following definitions:

Small pelagic fish (SP): pelagic fish with maximum length < 60cm;

Large pelagic fish (LF): pelagic fish with maximum length > 60cm;

Groundfish (G): all shelf-associated demersal fish;

Deep water fish (DF): bottom-associated fish found in off shelf waters (typically deeper than 500 m);

Unspecified fish (UF);

Pelagic crustaceans (PC);

Benthic crustaceans (BC): all bottom-associated crustaceans;

Unspecified crustaceans (UC);

Unspecified molluscs (UC);

Squid (S): all squid;

Bivalves (B): all bivalves.

We used a similar process (identifying the top ranking gear types in terms of catch and reporting those that collectively comprise ≥50% of the effort) to identify the dominant gear

types for each FAO Area (Table 1). Tanocet et al. (2019) do not provide gear type data for the Southern Ocean (FAO Areas 88, 48, 58), but we were able to characterise Southern Ocean catch by gear type, providing data that are comparable to the majority of other FAO Areas. The dominant Southern Ocean fisheries use either longlines to target toothfish (FAO Area 58 & 88) or trawls to target Antarctic krill and mackerel icefish (FAO Area 48, Table 1). In the case of the Northeast Atlantic, gear type data is presented in terms of percentage of fishing hours rather than percentage of catch (Taconet *et al.*, 2019). Table 1 presents these data, which suggest that trawls are the main fishing gear in the Northeast Atlantic, comprising more than 70% of fishing hours. It is therefore plausible that trawls are also the main fishing gear by catch, although the two metrics are not strictly comparable.

Table S1. Classification of dominant taxa recorded in the FAO Global Capture Production database (FAO, 2019) into the catch categories used in the current study.

Taxa	Category	Abbreviation
American lobster	Benthic crustacean	BC
Argentine red shrimp	Benthic crustacean	BC
Northern prawn	Benthic crustacean	BC
Marine crustaceans nei	Unspecified crustaceans	UC
Antarctic toothfish	Deep water fish	DF
Blue grenadier	Deep water fish	DF
Bombay-duck	Deep water fish	DF
Patagonian grenadier	Deep water fish	DF
Patagonian toothfish	Deep water fish	DF
Alaska pollock(=Walleye poll.)	Groundfish	G
Argentine hake	Groundfish	G
Atlantic cod	Groundfish	G
Cape hakes	Groundfish	G
Croakers, drums nei	Groundfish	G
Hairtails, scabbardfishes nei	Groundfish	G
Largehead hairtail	Groundfish	G
Pacific cod	Groundfish	G
Whitemouth croaker	Groundfish	G
Seerfishes nei	Large pelagic fish	LP
Skipjack tuna	Large pelagic fish	LP
Yellowfin tuna	Large pelagic fish	LP
Akiami paste shrimp	Pelagic crustacean	PC
Antarctic krill	Pelagic crustacean	PC
Natantian decapods nei	Pelagic crustacean	PC
Anchoveta(=Peruvian anchovy)	Small pelagic fish	SF
Atlantic chub mackerel	Small pelagic fish	SP
Atlantic herring	Small pelagic fish	SP
Atlantic mackerel	Small pelagic fish	SP
Atlantic menhaden	Small pelagic fish	SP
Bonga shad	Small pelagic fish	SP
California pilchard	Small pelagic fish	SP
Cape horse mackerel	Small pelagic fish	SP
Capelin	Small pelagic fish	SP

Clupeoids nei	Small pelagic fish	SP
European anchovy	Small pelagic fish	SP
European pilchard(=Sardine)	Small pelagic fish	SP
European sprat	Small pelagic fish	SP
Gulf menhaden	Small pelagic fish	SP
Hilsa shad	Small pelagic fish	SP
Indian mackerel	Small pelagic fish	SP
Indian mackerels nei	Small pelagic fish	SP
Indian oil sardine	Small pelagic fish	SP
Jack and horse mackerels nei	Small pelagic fish	SP
Japanese anchovy	Small pelagic fish	SP
Pacific anchoveta	Small pelagic fish	SP
Pacific chub mackerel	Small pelagic fish	SP
Pacific thread herring	Small pelagic fish	SP
Round sardinella	Small pelagic fish	SP
Sardinellas nei	Small pelagic fish	SP
Scads nei	Small pelagic fish	SP
Short mackerel	Small pelagic fish	SP
Southern African anchovy	Small pelagic fish	SP
Southern blue whiting	Small pelagic fish	SP
Marine fishes nei	Unspecified fish	UF
American cupped oyster	Bivalve	B
American sea scallop	Bivalve	B
Striped venus	Bivalve	B
Argentine shortfin squid	Squid	S
Cephalopods nei	Squid	S
Various squids nei	Squid	S
Wellington flying squid	Squid	S
Marine molluscs nei	Unspecified molluscs	UM

3. Indicators of carbon sink intensity and fishing intensity

There are no data available that provide reliable measures of either the ultimate benefit of the ocean biological carbon sink (the rate of deep carbon sequestration) or the ecosystem impact of fishing at the spatial resolution of our analysis. We therefore used available indicator variables. Here we briefly discuss the relationship between our indicator variables and the variables that they indicate.

The phytoplankton-driven carbon sink initially depends on the amount of primary production in the euphotic zone, and the proportion of this which gets exported out of the euphotic zone (i.e. export efficiency). Unlike carbon sequestration (or transfer efficiency through the mesopelagic) there is a consensus about how export efficiency varies across the global oceans, and hence we deem this the most appropriate metric of the potential carbon sink on a global scale. However, exported sinking carbon then attenuates rapidly through the mesopelagic zone, being consumed by heterotrophs such as zooplankton and bacteria, meaning only ~ 15 % reaches the deep ocean (i.e. > permanent thermocline, in deep water masses or the sediments) (Turner, 2015; Weber *et al.*, 2016). How this varies across the

global oceans is not clear, with some meta-analyses suggesting an inverse relationship with temperature (Henson *et al.*, 2012) and others a positive relationship (Marsay *et al.*, 2015). In addition, the varying depth of the permanent thermocline needs to be considered (Palevsky and Doney, 2018). To realise global maps of deep ocean phytoplankton-derived carbon sequestration more needs to be understood on the controls of mesopelagic carbon attenuation (Cavan *et al.*, 2019).

Some of the identified routes of fishery disturbance to the carbon sink (such as removing low-mid trophic level pellet-producing species) are directly related to catch (i.e. the amount of biomass removed). Others (e.g. sediment disturbance) are more directly related to effort (i.e. the amount of time spent fishing). Global effort data are available at the spatial resolution of our analysis (Fig. 2) whereas available catch data are generally aggregated at coarser spatial scales, including the FAO Areas. The catch achieved per unit effort depends on the local biomass of fish and the efficiency of the fishing gear. Effort is therefore an imperfect indicator of catch. Nonetheless it is well correlated with catch at the scale of FAO Areas (Fig S2). An important caveat with the effort data is that the ecosystem impact per unit fishing time varies with multiple factors including gear type (which is considered in our discussion), where it is deployed (above or below the thermocline, in the water column or on the sea bed), size of vessel, and other technologies used (e.g. fish finders and fish aggregation devices). Thus our analysis, based on effort data, provides an indication, rather than a definitive identification, of the potential spatial distribution of fishery impacts on the carbon sink and areas of high risk.

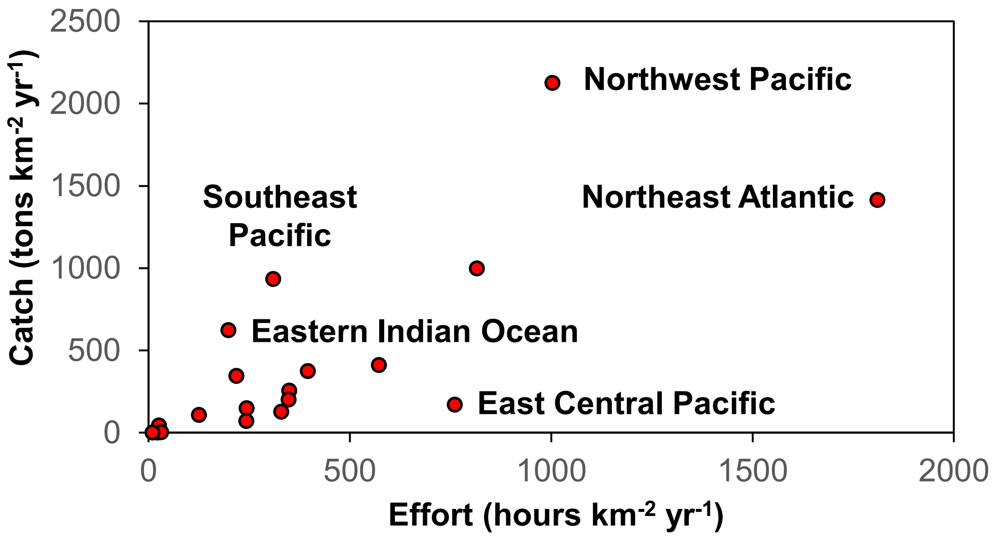

Fig. S2. *The relationship between our indicator of fishing impact (effort) and direct impact on target species biomass (catch) at the scale of FAO Areas (Pearson correlation coefficient=0.75, P<0.001), with key outliers labelled. The non-area-adjusted data (catch in tons yr^{-1} versus effort in hours yr^{-1}) are also correlated (Pearson correlation coefficient=0.72, P<0.001).*

References

Cavan, E. L., Laurenceau-Cornec, E. C., Bressac, M., and Boyd, P. W. 2019. Exploring the ecology of the mesopelagic biological pump. Progress in Oceanography: 102125. http://www.sciencedirect.com/science/article/pii/S0079661119300370.

DeVries, T., and Weber, T. 2017. The export and fate of organic matter in the ocean: New constraints from combining satellite and oceanographic tracer observations. Global Biogeochemical Cycles, 31: 535–555. Wiley Online Library.

Dunne, J. P., Armstrong, R. a., Gnanadesikan, A., and Sarmiento, J. L. 2005. Empirical and mechanistic models for the particle export ratio. Global Biogeochemical Cycles, 19: GB4026.

FAO. 2019. FAO yearbook. Fishery and Aquaculture Statistics 2017. Rome.

Henson, S. A., Sanders, R., Madsen, E., Morris, P. J., Le Moigne, F., and Quartly, G. D. 2011. A reduced estimate of the strength of the ocean's biological carbon pump. Geophysical Research Letters, 38: L04606.

Henson, S. A., Sanders, R., and Madsen, E. 2012. Global patterns in efficiency of particulate organic carbon export and transfer to the deep ocean. Global Biogeochemical Cycles, 26: n/a-n/a. http://doi.wiley.com/10.1029/2011GB004099 (Accessed 18 October 2018).

Kroodsma, D. A., Mayorga, J., Hochberg, T., Miller, N. A., Boerder, K., Ferretti, F., Wilson, A., *et al.* 2018. Tracking the global footprint of fisheries. Science, 359: 904–908. American Association for the Advancement of Science.

Laws, E. A., Sa, E. D., and Naik, P. 2011. OCEANOGRAPHY : METHODS Simple equations to estimate ratios of new or export production to total production from satellite-derived estimates of sea surface temperature and primary production: 593–601.

Marsay, C., Sanders, R., Henson, S., Pabortsava, K., Achterberg, E., and Lampitt, R. 2015. Attenuation of sinking particulate organic carbon flux through the mesopelagic ocean. Proceedings of the National Academy of Sciences, 12: 1089–1094.

Palevsky, H. I., and Doney, S. C. 2018. How Choice of Depth Horizon Influences the Estimated Spatial Patterns and Global Magnitude of Ocean Carbon Export Flux. Geophysical Research Letters, 45: 4171–4179. John Wiley & Sons, Ltd. https://doi.org/10.1029/2017GL076498.

Taconet, M., Kroodsma, D. A., and Fernandes, J. A. 2019. Global Atlas of AIS-based fishing activity - Challenges and opportunities. Rome.

Turner, J. T. 2015. Zooplankton fecal pellets, marine snow, phytodetritus and the ocean's biological pump. Progress in Oceanography, 130: 205–248. Elsevier Ltd. http://dx.doi.org/10.1016/j.pocean.2014.08.005.

Watson, R. A. 2017. A database of global marine commercial, small-scale, illegal and unreported fisheries catch 1950–2014. Scientific data, 4: 1–9. Nature Publishing Group.

Weber, T., Cram, J. A., Leung, S. W., DeVries, T., and Deutsch, C. 2016. Deep ocean nutrients imply large latitudinal variation in particle transfer efficiency. Proceedings of the National Academy of Sciences, 113: 8606–8611. http://www.pnas.org/lookup/doi/10.1073/pnas.1604414113.
